# The effectiveness of the Dutch Meaningful Roles program in children: a study protocol for a cluster randomized controlled study

**DOI:** 10.1186/s12889-023-16362-8

**Published:** 2023-07-27

**Authors:** Amanda W. G. van Loon, Tessa M. L. Kaufman

**Affiliations:** Utrecht University. Child and Adolescent Studies, Heidelberglaan 1, 3584 CS Utrecht, The Netherlands

**Keywords:** Classroom-based intervention, Children, Prosocial classroom climate, Wellbeing, Self-determination-theory

## Abstract

**Background:**

A positive, prosocial classroom climate is associated with improved social competence and academic achievement, as well as with decreased internalizing problems and antisocial behavior in children. It is expected that motivation to behave prosocially is needed to achieve a prosocial climate in the classroom, and that such motivation can be enhanced through three components of self-determination theory (SDT): competence, relatedness, and autonomy. The goal of this protocol is to describe the design of a study aiming to evaluate the effectiveness of a classroom-based program based on SDT components promoting a prosocial classroom climate.

**Methods:**

A cluster randomized controlled trial (RCT) will be conducted to examine the effectiveness of the classroom-based program Meaningful Roles, aiming to improve prosocial classroom climate through increasing children’s intrinsic prosocial motivation, stimulated by increasing social autonomy, social competence, and social relatedness. A multi-informant (i.e., children, teachers, and school leaders) and multi-method (i.e., questionnaires and focus groups) approach will be used to assess primary outcomes (i.e., prosocial behavior, intrinsic (prosocial) motivation, social autonomy, social competence, and social relatedness) and secondary outcomes (i.e., school wellbeing, social position, bullying, victimization, and civic skills), as well as moderators (i.e., working elements, child, teacher, school, and program characteristics, and program integrity).

**Discussion:**

The current study will provide information on the effectiveness of a classroom-based program promoting a prosocial classroom climate. It is of crucial importance that the school environment can provide a positive, prosocial classroom climate in which children feel safe and can achieve optimal social and academic competence and wellbeing.

**Trial registration:**

ClinicalTrials (NCT05891067).

## Background

A positive, prosocial classroom climate is crucial in promoting children’s school and psychological wellbeing, as well as in optimizing an effective learning environment [1, 2]. Previous research demonstrated that bullying perpetration and being victimized are less common in classrooms with a positive, caring, and supportive climate [3, 4]. Although classroom climate can be conceptualized in many ways, we focus on the emotional classroom climate domain as it is considered to be most vital to other aspects of classroom climate [2]. Positive emotional classroom climate is characterized by a prosocial behavior classroom norm (both injunctive and descriptive), high levels of collaboration, high quality of teacher and peer interactions, as well as low level of conflicts [1, 5, 6].

What is needed to create a prosocial classroom climate? According to the self-determination theory (SDT), behaviors are only encouraged when children experience intrinsic motivation for these behaviors. Moreover, such motivation (and behavior) is stimulated when children perceive that their environment fulfills their three fundamental needs: for autonomy, competence and relatedness (i.e., how autonomous, competent and related they feel within a particular context) [7, 8]. Enhancement of the needs of autonomy (the feeling of being independent, being able to decide for oneself), competence (confidence in one’s abilities) and relatedness (sense of belonging, trust in others) enables, for instance, greater empathy toward others, better coping with conflict, internalization of a prosocial behavior classroom norm, and a caring classroom environment [8]. Consequently, this increases children’s motivation to behave prosocially [8]. According to the goal-framing theory, the prevailing group norm influences what behavior is supported and reinforced and what behavior is not [9]. To ensure that prosocial behavior becomes a persistent basis of the classroom climate, prosocial behavior should become the social norm in the classroom (i.e., normative goal). Maintaining such a norm occurs through perceiving prosocial attitudes and support by others, such as peers and teachers [9]. The current protocol describes a study that examines the effectiveness of a classroom-based program promoting a prosocial classroom climate in primary schools, stimulated by increasing STD components and prosocial intrinsic behavior (i.e., Dutch adaptation of Meaningful Roles [SterkWerk]).

The program is based on Meaningful Roles, an antibullying intervention program offering bullies prosocial alternatives for their behavior (by increasing autonomy and relatedness) [10]. Results demonstrated reduced aggressive and problem behaviors at one school (e.g., fewer fights, detentions) [10], suggesting the positive effects of promoting prosocial behavior. Other research also showed the effectiveness of such intervention programs. Prosocial behavior interventions were found to be effective in promoting prosocial behavior in children and adolescent samples [11–13], especially for programs that were designed to increase social competence instead of preventing problem behavior [11]. Additionally, one single-school pilot study demonstrated that a one-year classroom climate intervention (focused on mindfulness, prosocial behavior, self-curiosity, and self-acceptance) improved the classroom environment for both teachers and students [14]. Furthermore, meta-analyses demonstrated that classroom-based social and emotional learning interventions had small to large effects on improving social and emotional skills, positive attitudes, positive social behavior, and indicators of wellbeing (e.g., fewer conduct problems, less emotional distress) in children and adolescents [15–18]. In addition, recent meta-analyses showed that anti-bullying programs were effective in decreasing school-bullying perpetration and victimization in children and adolescents [19, 20], revealing larger effects for specific components (e.g., whole-school approach, classroom rules, information for parents, informal peer involvement) [21] and specific groups (i.e., younger and more heavily victimized participants) [20]. Additionally, a recent meta-analysis in youth and adult samples demonstrated that SDT-informed intervention programs were associated with positive changes in SDT constructs (i.e., autonomy, competence and relatedness), as well as positive changes in physical and psychological health [22].

In sum, there is both theoretical evidence and there are empirical suggestions that prosocial classroom climate can be improved through enhancing children’s motivation to behave prosocially by targeting SDT components. Moreover, empirical research on the effectiveness of school- or classroom-based programs in primary school demonstrated promising results for improving prosocial classroom climate, increasing prosocial behavior and wellbeing, and decreasing antisocial behavior. However, previous knowledge can be extended in several ways. First, about half of the studies performed a (cluster) RCT, which means that in non-randomized studies there might be confounding factors. Second, most meta-analytic reviews and studies included several age cohorts (e.g., from kindergarten through high school, 4–18 years, from children to adults), not only primary school students, which precludes strong conclusions about this specific age group. Third, most studies were conducted in the United States, precluding conclusions about non-US samples. Hence, there is still not sufficient evidence for the effectiveness of classroom-based programs promoting prosocial behavior, specifically for programs based on STD components in primary school students. In addition, little is known about potential moderators of program effectiveness and the working elements of such programs.

Moderators are characteristics that are likely to affect the effectiveness of intervention programs, such as child, teacher, school, and program characteristics. Child characteristics that may affect program effectiveness include demographic characteristics (e.g., age, gender, ethnic identity background) and other factors (e.g., severity of problems or popularity of bullies at baseline). For instance, previous meta-analytic reviews showed that school-based interventions with older adolescents [23], a higher proportion of female adolescents [23, 24], and a greater proportion of ethnic minority youth [1, 23] were more effective. Furthermore, previous research showed larger program effects for children with more problem behavior before the start of the intervention [13, 25], while lower program effects were found for bullies with a high popularity at baseline [26].

Teacher characteristics that may affect program effectiveness include demographic characteristics (e.g., age, gender, ethnic identity) and other factors (e.g., years of experience). Previous meta-analytic studies examining the associations between student–teacher relationships and school adjustment showed that teachers’ gender and ethnicity moderated these associations [27, 28]. For example, female teachers might be able to better implement socioemotional programs, due to the traditional gender role of females as effective caretakers of child development [27]. In addition, experienced or older teachers might be more effective in implementing the program because they have more knowledge and have acquired greater skill. Indeed, previous research showed that teaching experience is positively related to student outcomes (e.g., achievement, motivation) [29]. School characteristics, such as years of experience with a social safety program (e.g., KiVa), school or class size, or location may moderate the effectiveness of the program. In line with this reasoning, if schools already have (much) experience with implementing social safety programs, it is likely that teachers at those schools may be more effective in implementing the current program. Furthermore, it is likely that teachers give students more individual attention in smaller classes, which might be important during classroom-based intervention programs and hence, may result in stronger effects. Program effectiveness may also differ based on location, as a previous meta-analysis examining the effectiveness of family interventions demonstrated smaller effects for urban settings compared to nonurban settings [30].

Program characteristics that may affect program effectiveness include program integrity and working elements. Program integrity, whether the intervention is implemented as originally planned, is important to report and to investigate as moderator of effectiveness, as it is necessary to draw conclusions about an intervention program. Negative or nonsignificant results might be due to incorrect program implementation, and not because of an ineffective program [31]. In addition, whether the working elements (i.e., meaningful roles, classroom meetings, and compliments) were implemented and how often may influence program effectiveness. Indeed, previous literature demonstrated that better implementation of school-based prevention programs is associated with more beneficial outcomes [15, 32].

### Current study

**Fig. 1:**
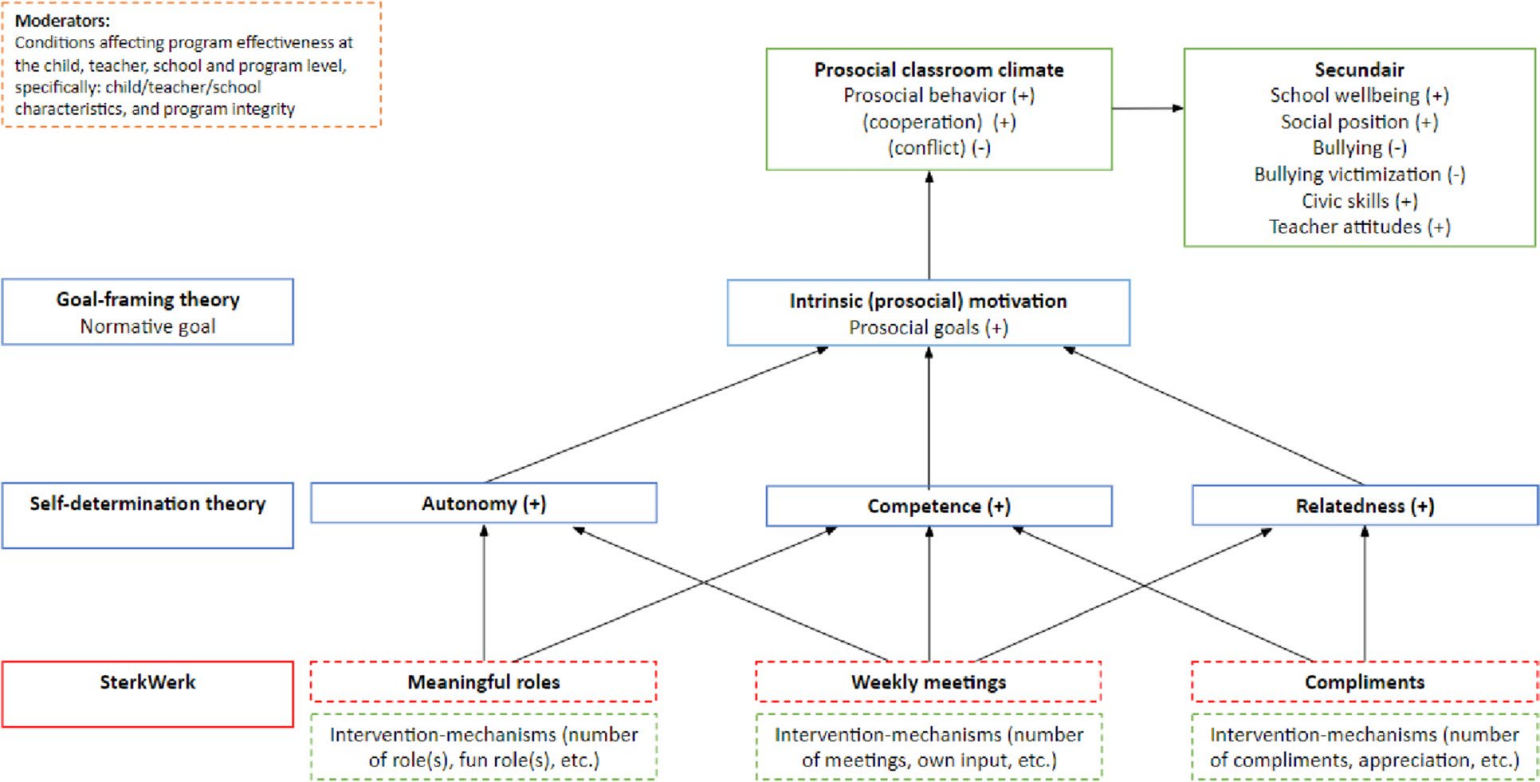
Conceptual model of the research design

In sum, the current protocol describes a study that will be conducted to examine the effectiveness of a classroom-based program promoting a prosocial classroom climate (i.e., Meaningful Roles). Figure 1 presents the conceptual model of the research design. A cluster RCT will be performed to investigate if the program results in more prosocial classrooms (e.g., more prosocial behavior, more cooperation, less conflict). The program will be compared to a control condition. We will use a multi-informant (children, teachers, and school leaders) and multi-method (questionnaires and focus groups) design. As illustrated in Fig. 1, it is expected that Meaningful Roles will increase children’s social autonomy, social competence, and social relatedness, resulting in an increase in intrinsic (prosocial) motivation and a prosocial classroom climate (i.e., increased prosocial behavior, positive teacher attitudes, civic skills, and school wellbeing, and reduced bullying and bullying victimization). In addition, the second aim is to examine moderators of effectiveness. This is important because it is likely that Meaningful Roles is not equally effective across child, teacher, school, and program characteristics (i.e., program integrity). The final aims of this study are to evaluate the working elements of Meaningful Roles (i.e., meaningful roles, classroom meetings, and compliments) and to evaluate how the program is experienced by teachers and school leaders.

## Methods

### Design

**Fig. 2:**
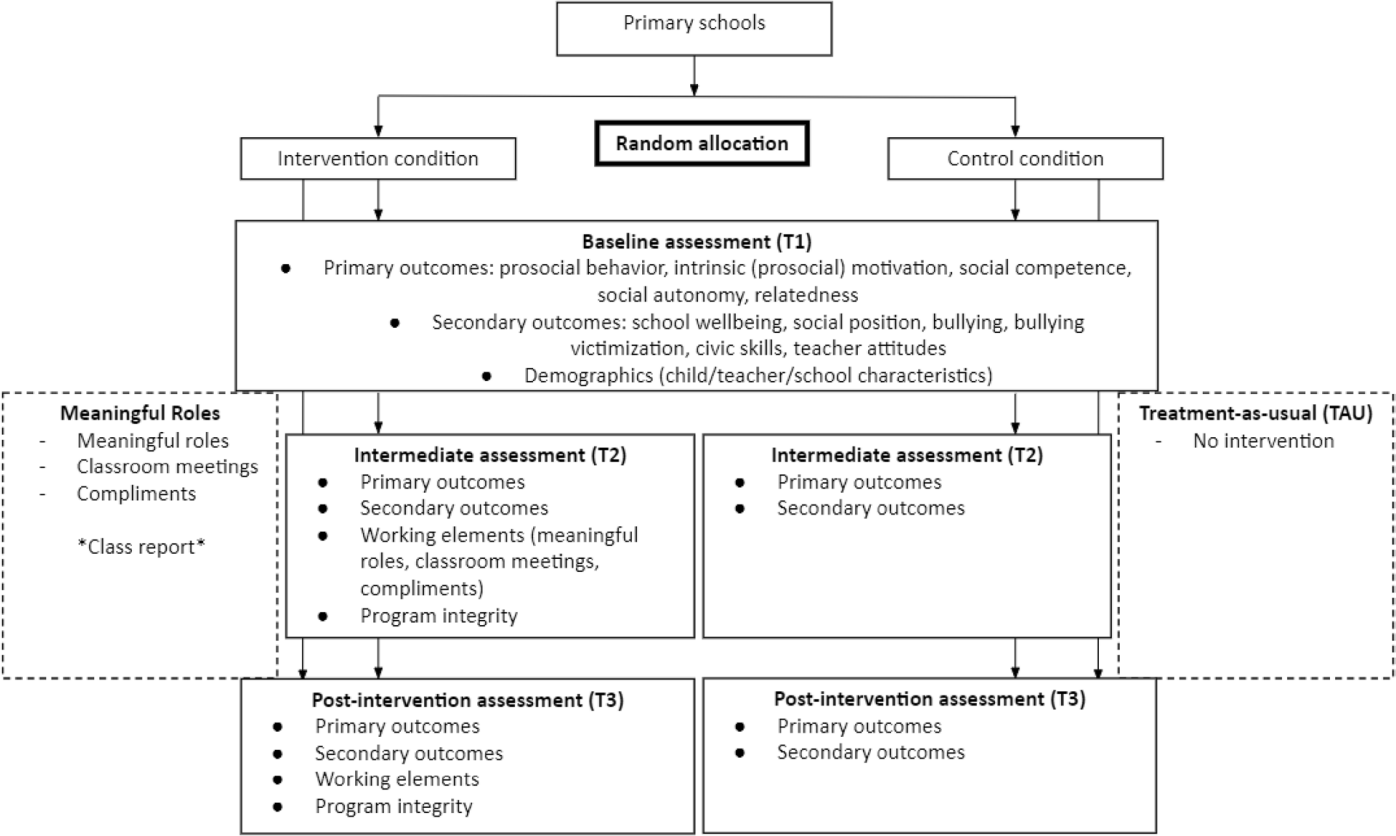
Flow chart of the research design

A cluster RCT will be conducted. Schools will be randomized (stratified for number of participating classrooms) into the intervention condition in which the program starts immediately or into a control condition that receives the program one school year later (see Fig. 2), using an online randomization tool [33] in a 1:1 ratio. We will recruit a diverse sample of children from primary schools in the Netherlands.

### Data collection

Online questionnaires assessing demographics (e.g., age, gender), primary outcomes (i.e., prosocial behavior, intrinsic prosocial motivation, social competence, social autonomy, and social relatedness) and secondary outcomes (i.e., school wellbeing, social position, and antisocial behavior) will be completed by children prior to the start (T1) of the program. Halfway through the program (T2) and after completion of the program in the intervention condition (T3), children complete questionnaires about program content (i.e., working elements) in addition to questionnaires assessing primary and secondary outcomes. Teachers complete questionnaires about demographics (e.g., age, years of experience), attitudes, and civic skills at T1 and questionnaires about attitudes, civic skills, program content (to assess program integrity), and program evaluation at T2 and T3. Additionally, one teacher from each school will participate in the focus groups (at T1, T2, and T3), to evaluate the implementation of the program (i.e., qualitative information). Table 1 presents an overview of the instruments at each assessment point.

**Table 1:** Overview of the variables’ instruments and sources

Outcome	Variable name	Instrument	Time of measurement	Variable type	Source
Primary outcomes					
Prosocial behavior	Injunctive norm	Developed for this study	T1, T2, T3	Outcome	Child
	Prosocial behavior (individual)	Based on the Strengths and Difficulties questionnaire (SDQ), subscale prosocial behavior	T1, T2, T3	Outcome	Child
	Prosocial behavior (according to classmates)	Sociometric nominations	T1, T2, T3	Outcome	Child
	Cooperation in the classroom	Classroom Peer Context Questionnaire (CPCQ), subscale cooperation	T1, T2, T3	Outcome	Child
	Conflict in the classroom	Classroom Peer Context Questionnaire (CPCQ), subscale conflict	T1, T2, T3	Outcome	Child
Intrinsic (prosocial) motivation	Social goals	Developed for this study	T1, T2, T3	Outcome	Child
Social competence	Self-esteem	Developed for this study, based on de Rosenberg Self-Esteem Scale	T1, T2, T3	Outcome	Child
Social autonomy	Social autonomy and responsibility	Developed for this study, based on the School as a Caring Community Profile – II and Child Development Project [34]	T1, T2, T3	Outcome	Child
Social relatedness	Comfort and cohesion	Classroom Peer Context Questionnaire (CPCQ), subscale comfort and cohesion	T1, T2, T3	Outcome	Child
Secondary outcomes					
School wellbeing	School belonging and safety	School belonging and safety	T1, T2, T3	Outcome	Child
Social position	Injunctive norm (nominations)	Sociometric nominations	T1, T2, T3	Outcome	Child
Bullying and victimization	Bullying and victimization	Olweus Bully-Victim questionnaire and sociometric nominations	T1, T2, T3	Outcome	Child
Civic skills	Civic skills	Based on the Vragenlijst Burgerschap Meten	T1, T2, T3	Outcome / moderator	Teacher
Working elements					
	Meaningful roles	Developed for this study	T2, T3	Outcome / moderator	Child, teacher
	Classroom meetings	Developed for this study	T2, T3	Outcome / moderator	Child, teacher
	Compliments	Developed for this study	T2, T3	Outcome / moderator	Child, teacher
Moderators					
Child characteristics	Demographics	Developed for this study	T1	Moderator / covariate	Child
Teacher characteristics	Demographics	Developed for this study	T1	Moderator / covariate	Teacher
	Attitudes	Developed for this study	T1, T2, T3	Moderator / outcome	Teacher
Program characteristics	Program integrity	Developed for this study	T2, T3	Moderator / covariate	Teacher, school leader

The design of this study has been approved by the Ethical Committee of Utrecht University (number 23–0082) and is registered in the ClinicalTrials registry (NCT05891067).

### Study sample

We aim to include *N* = 42 schools to ensure there is enough power for the analyses (*N* = 21 schools for both the intervention and waitlist control condition). This sample size is sufficient to examine the effectiveness of Meaningful Roles and potential moderators, with a power of 0.80, an alpha of 0.05 and a medium effect size of 0.21 [18].

Participants are children from the upper grades of primary school (grades 5–8) in the Netherlands, their teachers (who will implement the program) and school leaders. The children will mostly be between 8 and 12 years old.

### Recruitment

Primary schools will be approached in person, via email and/or via telephone (mainly based on contacts of the intervention and research team). If schools show interest in participating in the Dutch adaptation of Meaningful Roles, they will receive (written) information about the project and what participation entails. Schools are also invited to an online orientation meeting. In this meeting the intervention and research team will clarify information about the program and research study, as well as answer questions from schools. If schools agree to participate, they will sign a cooperation form and will be randomized into the intervention or waitlist control condition. Parents/caregivers of children will be informed about the project (via mail, information evenings, telephone) by schools and will be asked to give active informed consent for participation of their child in the study (on paper or digital). Children aged 12 years or older will be asked for active informed consent themselves before they start the online questionnaire, in addition to their parents/caregivers. All children will be notified that participation is voluntary and that they can stop at any moment. Teachers and school leaders will be asked for active informed consent for their own participation in the study via a digital consent form, right before they start the online questionnaire. Children without permission/consent do not receive the research questionnaire, but they do receive the Meaningful Roles program.

### Dutch Meaningful Roles program

The Dutch adaptation of Meaningful Roles is a classroom-based program implemented by teachers. Teachers receive two three-hour training sessions from trained and experienced educational professionals. Meaningful Roles is an approach (throughout the schoolyear) for primary school groups 5 to 8 (children between 8–12 years old). The aim of Meaningful Roles is to improve the classroom climate and mutual relationships in the group, in a positive, prosocial way.

The program consists of three main components: meaningful roles, classroom meetings, and compliments (see Fig. 1 for the conceptual model). With regard to the roles, children are given specific roles to actively work on autonomy and responsibility, competence, and prosocial behavior. These roles, such as newsreader or plant caretaker, are prosocial in nature, because children help one or more children or are considerate of other children in the class. Moreover, roles give children autonomy and responsibility, as the children are free to perform the role as they see fit. The roles are aimed to optimize the strengths and talents of each child, through giving children the experience that they can contribute something that increases their confidence in their own abilities. During the weekly classroom meetings, children take the leading role in discussions about the classroom functioning and agreements: they discuss how they feel and what the atmosphere is like in the classroom. This ensures interaction and connection with each other and stimulates children’s feeling of competence, autonomy and responsibility in social classroom processes. Last, children learn how to give and receive effective compliments and practice with doing so on a structural basis, which strengthens trust in others and reinforces confidence in one’s own abilities.

### Control condition

The control condition will not receive any material or training concerning Meaningful Roles during the implementation of the program in the intervention condition (treatment-as-usual, TAU). Schools in the control condition are given the option to receive the materials and training to implement the intervention at a later stage, after the data collection is finished.

### Instruments

#### Primary outcomes

*Prosocial behavior* will be measured by several instruments measuring different domains (i.e., injunctive norm, individual prosocial behavior, sociometric nominations, cooperation in the classroom and conflict in the classroom).

Prosocial behavior as an injunctive norm (i.e., children’s attitudes towards prosocial behavior that are on average held in the classroom; [35]) will be assessed with three items specifically developed for this study (i.e., What can make you popular in your class?: “Helping each other”, “Being nice to each other” or “Working together”), rated on a 3-point scale from 1 (*not true*) to 3 (*definitely true*). Higher scores reflect a higher injunctive norm of the group towards prosocial behavior.

Individual prosocial behavior will be measured with the subscale prosocial behavior of the Strengths and Difficulties Questionnaire (SDQ), Dutch version [36], adapted for the current study. Four items are used (e.g., “I try to be nice to my classmates”), rated on a 4-point scale from 1 (*never*) to 4 (*always*). The SDQ-Dutch has sufficient psychometric properties in a children sample, with a Cronbach’s alpha of 0.78 for the total scale and 0.62 for the prosocial subscales [37]. Higher scores reflect more prosocial behavior.

Prosocial behavior according to children’s classmates will be measured with sociometric nominations. For each question, children see a list with the names of all classmates and select the classmates that fit the question best according to them [38]. Two items are used (i.e., “Which classmates help you with assignments (for example with math or language)?” or “Which classmates help you with problems (for example, when you are sad)?”). For each child, the number of received nominations was divided by the number of possible nominations (i.e., number of classmates), resulting in a proportion score for prosocial relations [39].

Cooperation and conflict in the classroom will be measured with the Classroom Peer Context Questionnaire (CPCQ), subscales cooperation and conflict, respectively [38, 40], rated on a 4-point scale ranging from 1 (*not true*) to 4 (*completely true*). The cooperation subscale consists of four items assessing the extent to which children experience positive behavior (e.g., “In this classroom, children help each other”). The conflict subscale consists of four items assessing the extent to which children experience negative behavior. For the current study, two items are used (i.e., “In this classroom, children are unkind to each other” and “In this classroom, children bully each other”). The CPCQ has adequate psychometric properties in a children sample, with a Cronbach’s alpha of 0.79 and 0.83 for the cooperation and conflict subscale, respectively [38]. Higher scores on the cooperation subscale reflects more positive behavior, while for the conflict subscale it reflects more negative behavior.

*Intrinsic (prosocial) motivation* will be measured with four items specifically developed for this study (e.g., “I think it is important to help someone” or “I want to know how to be a good friend”), rated on a 3-point scale ranging from 1 (*not true*) to 3 (*definitely true*). Higher scores reflect more intrinsic (prosocial) motivation.

*Social competence* will be measured with items based on the Rosenberg Self-Esteem Scale (RSES), Dutch version [41, 42]. Five items are used (e.g., I think positively about myself), rated on a 3-point scale ranging from 1 (*not true*) to 3 (*definitely true*). The Dutch RSES showed high internal consistency in adults [41] and sufficient psychometric properties in adolescent samples [43, 44]. Higher scores reflect more perceived social competence.

*Social autonomy* will be measured with items based on the School as a Caring Community Profile-II (SCCP-II; (Lickona and Davidson. School as a caring community Profile II, unpublished)) and the Child Development Project [34, 45]. Five items are used (e.g., “In this classroom, children help with making group appointments” or “In this classroom, you can choose how you want to do something”), rated on a 4-point scale ranging from 1 (*never*) to 4 (*always*). Higher scores reflect more social autonomy.

*ocial relatedness* will be measured with items of the Classroom Peer Context Questionnaire (CPCQ), subscales comfort and cohesion [38, 40], rated on a 4-point scale ranging from 1 (*not true*) to 4 (*completely true*). The comfort subscale consists of four items assessing the extent to which children feel at ease in the classroom (e.g., “In this classroom, I can be myself”). The cohesion subscale consists of four items assessing the extent to which the children in the classroom spend time together. For the current study, two items are used (i.e., “In this classroom, everyone likes each other” and “In this classroom, everyone belongs to the group”). The CPCQ has adequate psychometric properties in a children sample, with a Cronbach’s alpha of 0.83 and 0.68 for the comfort and cohesion subscale, respectively [38]. Higher scores on both subscales reflect more positive behavior.

#### Secondary outcomes

*School wellbeing* will be measured with eight items specifically developed for this study, assessing school belonging (7 items; e.g., “I like going to school” or “It is fun and nice in the classroom”), rated on a 4-point scale ranging from 1 (*never*) to 4 (*always*) and school safety (i.e., “I feel safe in and around school”), rated on a 5-point scale ranging from 1 (*very unsafe*) to 7 (*very safe*). Higher scores reflect positive school wellbeing.

*Social position* will be measured with sociometric nominations. For each question, children see a list with the names of all classmates and select the classmates that fit the question best according to them [38]. Questions are related to kindness (i.e., “Which classmates do you like?”, leadership (i.e., “Are there any children in your class who are leaders?”), unkindness (i.e., “Which classmates do you not like at all?”), popularity (i.e., “Which children in your class are popular?”), and best friends (i.e., “Which classmates are your best friends?”). For each child, the number of received nominations was divided by the number of possible nominations (i.e., number of classmates), resulting in a proportion score for each social position (i.e., five proportion scores) [39].

*Bullying and victimization* will be measured by assessing bullying and victimization with a selection of items of the Olweus Bully-Victim Questionnaire, Dutch version (OBVQ; [46]) and sociometric nominations. The OBVQ consists of items related to bullying, bullying victimization, and the perceived behavior and attitudes of the teacher. Two questions are examined, related to bullying (“How often have you bullied other children in the past few months?”) and bullying victimization (“How often have you been bullied in the past few months?”). Responses are rated on a 5-point Likert scale: *I did not bully/I was not bullied*, *only once or twice*, *two or three times a month*, *about once a week*, and *several times a week*. Seven additional questions relate to bullying victimization, and ask about behaviors that are associating with bullying (e.g., “I was called names, ridiculed or laughed at” or “I was threatened or forced to do things I did not want to do”), rated on 5-point Likert scale ranging from 1 (*not at all*) to 5 (*several times a week*). One additional item asks where the child is bullied (e.g., “In the hall or corridor”, “During gym class” or “In another place”). Three items relate to the perceived behavior and attitudes of the teacher (i.e., “Has your teacher ever mentioned bullying during class?”, with answer options: no, once, 2–4 times, 5–8 times, more than 8 times’, “What does your teacher think about bullying?”, with answer options: it is a good thing, it does not matter, I do not know, it is a bad thing, it is a very bad thing, and “What can your teacher do to stop bullying?”, with answer options: nothing, very little, a little, a lot, very much). Answer options will be recalculated so that higher scores reflect more bullying, victimization, and negative attitudes/behavior of the teacher. The OBVQ showed adequate psychometric properties in children samples, with a Cronbach’s alpha above 0.80 [47–49]. Regarding sociometric nominations, ten questions related to bullying victimization and one question related to bullying will be asked to children (e.g., “Who starts bullying you”, “Who will participate if you are being bullied?” or “Which classmates help you when you are being bullied?”). For each question, children see a list with the names of all classmates and select the classmates that fit the question best according to them [38]. For each child, the number of received nominations was divided by the number of possible nominations (i.e., number of classmates), resulting in one proportion score for victimization (i.e., sum of all ten items) and one for bullying [39].

*Civic skills* will be assessed at all three timepoints, for teachers only. Specific questions were developed for this study, based on the questionnaire measuring citizenship ([Vragenlijst Burgerschap Meten]; [50]). Four domains will be assessed by asking teachers how the children in their class: 1) act democratically (e.g. “accept decisions made by the whole class (majority decisions)”, 2) act socially responsible (e.g., “stand up for each other”), 3) dealing with conflicts (e.g., “consider the other person in a conflict when looking for a solution”, and 4) dealing with differences (e.g., “are curious about what other children are good at (qualities)”. Higher scores reflect more (positive) civic skills in the classroom.

*Teacher attitudes* will be assessed at all three timepoints, with specific questionnaires developed for this study. Teachers are asked what they find important in their job as a teacher to stimulate, for example: “that children help each other in the classroom”. Higher scores reflect a positive attitude of stimulating prosocial behavior and STD components.

#### Moderators

*Child, teacher, and school characteristics* will be collected at baseline. Child characteristics include age, gender, romantic attraction (i.e., possibility of falling in love with a boy/girl), grade, and ethnic identity. Teacher characteristics include age, gender, years of experience (as a teacher in general, and as a teacher at the specific school) and experience with implementing social safety programs, as well as attitudes of the teachers. School characteristics include location, number of participating grades and students in the classrooms, and years of experience with a social safety program (e.g., KiVa).

*Working elements* will be assessed halfway through the program (T2) and after completion of the program (T3), with specific questions developed for this study. Children and teachers fill in questionnaires related to the three main components of the program: 1) meaningful roles (e.g., “How much did you like the role(s)”, “Which roles were the most popular?”, respectively), 2) classroom meetings (e.g., “The weekly meetings were done independently by the class”, “How often has a meeting been held?”, respectively), and 3) compliments (e.g., “I have received compliments from my classmates”, “How many times a week has attention been paid to the compliments?”, respectively). Higher scores reflect a more positive or greater adherence of the working elements.

*Program integrity* will be assessed halfway through the program and after completion of the program, with specific questions developed for this study. Teachers and school leaders fill in (open and closed) questionnaires related to the implementation of the program, divided into the domains training and material (e.g., “How often have you used the manual?”), child questionnaires (e.g., “Where there any questions that the children struggled with?”), time investment (e.g., “How did you feel about the time you had to implement the program properly?”), and global impression (e.g., “What is your general impression of the degree of success of the implementation of the program?”). Higher scores reflect a more positive or greater adherence of the program.

#### Teacher focus groups

In addition to the questionnaires, focus groups for teachers will be organized at T1, T2, and T3 to evaluate the implementation of Meaningful Roles. One delegate teacher per school will be asked to participate in these focus groups. Delegates can talk about their experiences with the intervention program and research, and are encouraged to make suggestions about important questions that should be included in the data collection waves for teachers and school leaders (at T2 or T3), in order to understand how feasible the implementation has been and what should be improved about the program. Semi-structured interviews will be used for the focus groups. The purpose of these focus groups is thus threefold: 1) information can be used as an indicator of the process evaluation (qualitative research information as an addition to the questionnaires) to understand strengths and difficulties in the execution of the intervention and evaluation of the research. This information can also help to understand why stronger or weaker effects may be found in different schools (i.e., interpretation of effects); 2) information can be used to give suggestions for improving the program in the future; and 3) information can be used to add any questions to the intermediate measurement (T2) and final measurement (T3) that, in the experience of the teachers, seem important to measure regarding the implementation (not the outcomes). These findings are used to clarify the findings in the context of the implementation process.

### Statistical analyses

Before the analyses, possible baseline (T1) differences in demographics and outcome variables (baseline assessment) between children in the intervention program and the control condition will be checked with ANOVA (continuous variables) and chi-square analyses (categorical variables). To investigate the effectiveness of the intervention program, regression analyses will be performed. The dependent variables are the outcome measures at intermediate (T2) or posttest (T3), condition is the predictor variable (1 = intervention, 0 = control), and baseline (T1), potential differences between the experimental and control group at T1, or intermediate measures (when dependent variables are the outcome measures at T2) are the covariates. The effect of potential moderators (categorial or continuous) on the outcomes will be examined by adding interaction effects to the models.

## Discussion

This study protocol presents the design of a cluster RCT to investigate the effectiveness of a classroom-based program, Meaningful Roles, to promote a more prosocial classroom climate in primary schools, through improving intrinsic motivation to behave prosocial, by enhancing SDT components (i.e., social autonomy, social competence, and social relatedness). This is important, because a positive, prosocial classroom climate is associated with an optimal learning environment, as well as positive school quality and wellbeing in children [1, 2]. Previous research showed improved prosocial classroom climate (e.g., increased prosocial behavior) in youth after receiving school- or classroom-based social-emotional focused programs (e.g., [11, 15]), as well as improved psychological health after receiving programs focused on STD components [22]. To overcome limitations of previous research, including suboptimal study design (e.g., non-randomization) and target group (e.g., US samples), we will examine a classroom-based approach for upper primary school students and use a cluster RCT to exclude confounding (e.g., allocation and selection bias) as much as possible. We will use a multi-informant (i.e., child, teacher, school leader) and multi-method (i.e., questionnaires and focus groups) approach. Moreover, we strive to use a large, diverse sample, that could allow us to examine potential moderators of program effectiveness (i.e., child, teacher, school, and program characteristics, i.e., working elements and program integrity).

There are several challenges in this study, such as the recruitment of a sufficient number of schools (and students) and the collaboration with multiple parties. First, it may be difficult to recruit sufficient schools with a diverse and representative sample of students and maintain the involvement of schools, teachers, students, and parents over the course of the study. Recruitment of schools may be difficult because we would preferably like schools to participate with all their upper classes (groups 5–8), which requires involvement and participation of multiple teachers (who all need to stand behind the program and research). To recruit schools, we will mainly try to contact schools who already have connections or collaborations with the research or implementation team (i.e., KiVa schools), making the first contact easier. These schools are located all throughout the Netherlands. As such, it is likely that diverse schools, with a diverse sample of students, will be represented in our study. Additionally, flyers will be distributed via mail and newsletters, and information meetings will be organized to give more information about the program and study participation.

Second, since participation of students requires active informed consent from their parents, it is crucial that all parents of participating classes are reached and are provided with clear information about study participation. Participating schools make sure that parents receive information about the project, and teachers will be responsible for contacting parents (e.g., face-to-face, mail, telephone). In order to make giving permission as easy as possible, parents can also give digital consent. Researchers and the contact person of the schools will be in constant contact to increase response rates. These precautions are expected to increase the likelihood of participation of students.

Third, another challenge in this study is the participation and involvement of multiple parties, including the researchers, the implementation team, trainers, schools, teachers, parents, and students. To strengthen the collaboration between parties, we will sign multiple agreement forms (e.g., data processing agreement, collaboration agreement). Moreover, we will organize regular meetings (between the research and implementation team) and give weekly updates about recruitment rates (to schools and teachers). Moreover, we will be available for schools for consultation at any time during the recruitment phase and implementation process. It is of crucial importance that we invest time in clear and timely communication with all parties in order to collaborate as effectively as possible. Furthermore, we try to work according to a standardized research protocol as much as possible, but will tailor it to the needs of the specific schools (e.g., regarding recruitment strategy, for instance using paper or digital informed consent forms). This way, we aim to avoid miscommunications and will ensure a positive and fruitful collaboration.

In sum, the current protocol describes a study that will examine the effectiveness of a classroom-based program, Meaningful Roles, in promoting a prosocial classroom climate in primary schools. It is of crucial importance that classrooms are a safe and positive environment for children to be in, to improve social competence and achievement, and prevent the development of mental health problems and dysfunction later in life.

## Data Availability

The data obtained in the current study will become publicly available after publication of the results on the main research questions. The data will be archived on the DANS Easy environment from the KNVB as agreed upon with NWO (open access policy). The corresponding author can be contacted for more information.
